# Possible Role and Function of AI Conversational Agents in Dialectical Behavior Therapy for Borderline Personality Disorder: Qualitative Interview Study

**DOI:** 10.2196/98840

**Published:** 2026-09-25

**Authors:** Niklas Liljedahl, Lilas Ali, Sophie I Liljedahl, Örjan Falk, Steinn Steingrimsson

**Affiliations:** 1 Institute of Neuroscience and Physiology, Sahlgrenska Academy, University of Gothenburg, Medicinaregatan 11, Gothenburg, Box 430, 405 30, Sweden, 46 0708257338; 2 Department of Psychiatry for Affective disorders, Region Västra Götaland, Sahlgrenska University Hospital, Gothenburg, Sweden; 3 Institute of Health and Care Sciences, Sahlgrenska Academy, University of Gothenburg, Gothenburg, Sweden; 4 Centre for Person-Centred Care (GPCC), University of Gothenburg, Gothenburg, Sweden

**Keywords:** artificial intelligence, AI, generative AI, attitude of health personnel, borderline personality disorder, chatbots, dialectical behavior therapy, mental health services, psychotherapy, qualitative research

## Abstract

**Background:**

Borderline personality disorder (BPD) is associated with substantial distress and a high risk for suicide. Individuals with BPD may be unable to access evidence-based treatments like dialectical behavior therapy (DBT). Artificial intelligence conversational agents (AI-CA) are increasingly discussed as scalable tools for mental health support, but little is known about how DBT clinicians understand the possible role of AI-CA in treatment.

**Objective:**

This study aimed to explore DBT psychologists’ perspectives on integrating AI-CA into DBT for BPD in the future.

**Methods:**

Seventeen psychologists in Sweden, each with at least 1 year of clinical DBT experience (mean 6.4 years, SD 5.6), participated in semistructured interviews as part of this qualitative study. Interviews were conducted in Swedish, transcribed verbatim, and analyzed using reflexive thematic analysis within a constructivist framework. Participants did not test a specific AI-CA.

**Results:**

Three main themes were developed from the data. The first main theme, “Who Are We in Therapy?” explored how participants defined AI-CA relationally, positioning it variously as a tool, team member, or supervisor, and a sometimes harmful competitor. How these positionings were configured shaped what AI-CA was seen as allowed to do. The second main theme, “The Stoic Helper,” captured how AI-CA was constructed as an extension of the ideal helper: available, competent, adaptable, and tireless, able to provide support in moments when human therapists could not or preferred not to be present. Participants’ hopes for what AI-CA could become often mirrored qualities they found difficult to sustain in their own clinical work. The third main theme, “The Well-Intended Accommodator,” captured concerns that AI-CA may reinforce dependency and function as a safety behavior by supporting reassurance-seeking rather than autonomy. A central concern was not whether AI-CA could generate validating responses, but whether it could know when validation supports change and when it becomes maladaptive accommodation (AI functional ambiguity).

**Conclusions:**

Perceived benefits mainly centered on accessibility and support for DBT skills generalization, whereas key concerns involved alliance disruption, reinforcement of behaviors that would ideally be targeted for change, dependency, and questions regarding responsibility in high-risk situations. Integrating AI-CA into DBT is not only a technical question but a relational and ethical one. How AI-CA is positioned in relation to the therapist, person in treatment, and team shapes which tasks are considered acceptable and what form integration can take. The findings highlight the need for implementation frameworks that account for relational dynamics, treatment-specific considerations, and AI functional ambiguity that may arise when AI-CA operates in complex therapeutic contexts.

## Introduction

### Background

Borderline personality disorder (BPD) is associated with significant functional impairment and profound distress [1]. It is characterized by intense, unstable interpersonal relationships marked by alternating idealization and devaluation, an unstable sense of self, affective instability, and a heightened risk of self-harm and suicidal behavior [1]. Yet access to specialist treatments such as dialectical behavior therapy (DBT) is limited [2], and there are implementation challenges [3,4]. Earlier digital interventions for BPD, predating AI-CA and commonly based on DBT or CBT, have shown mixed efficacy, with meta-analytic benefits for suicidal ideation and paranoia but not for overall symptom severity [5]. These DBT- and CBT-based tools nonetheless outperformed alternative approaches for suicidal ideation [5], suggesting that grounding digital support in an evidence-based framework matters. Interest in AI-enabled support for mental health is now growing and could help to extend evidence-based treatment to more people, though practical and ethical concerns complicate implementation [6,7]. For people with BPD, who experience interpersonal challenges, self-harm, and high risk for suicidal behavior [1], it is essential that we understand how to integrate AI into BPD treatment responsibly. This study provides an in-depth account of how DBT psychologists anticipate, imagine, and evaluate possible AI-CA integration within a DBT framework for BPD, which may also inform care for other populations with similar clinical needs.

### DBT, BPD, and Implementation Challenges

Standard DBT is an evidence-based treatment for BPD [8], delivered through individual therapy, group skills training (including homework assignment and review), phone coaching, and therapist consultation team [9,10]. These four modes serve DBT’s core functions: building patients’ skills, strengthening motivation to change, promoting generalization of skills to daily life, structuring the environment, and sustaining therapists’ capability and motivation [9,10]. DBT assumes that many difficulties reflect skills deficits [9,10]. Across treatment, therapists balance acceptance and change while helping patients act from Wise Mind, the integration of emotion mind and reasonable mind [9,10]. DBT combines validation of patients’ responses as understandable in context with contingency management to reinforce adaptive behavior [9,11]. Phone coaching supports real-time skills generalization before self-destructive behaviors occur [12,13].

For example, a patient who has just received a poor exam grade calls with high urges to self-harm. The therapist first validates the distress (“you worked so hard on that exam, and now you are anxious about failing the course”), then coaches a specific plan for the rest of the evening, and, when risk warrants it, also helps the patient reduce access to means. This real-time intervention reduces the risk of self-harm while helping the patient plan to cope with pain in more adaptive ways. Yet phone coaching is among the DBT components most challenging to implement and the least frequently delivered [3,4]. Perceived provider burden is a frequently cited reason for omission [14]. These implementation difficulties provide a clinical rationale for considering AI-CA in DBT, particularly for hard-to-sustain functions such as between-session skills generalization.

### Conversational Agents, AI, and Mental Health

AI-based conversational agents are increasingly being investigated as potentially scalable tools for mental health support, although evidence for their effectiveness and implementation in routine care remains limited [7,15,16]. Conversational agents relevant to mental health vary both in technical architecture and primary design intent. Architecturally, they range from rule-based systems using predefined scripts or decision trees, through hybrid systems combining natural language processing or machine learning with rule-based constraints, to generative AI systems, often large language model (LLM)-based, that produce more open-ended and contextually responsive dialogue [16-18]. In terms of design intent, agents may be developed specifically for therapeutic or wellness support [16], function primarily as social or relational companions [19], or be general-purpose generative AI systems that are not designed for mental health but are nevertheless used for emotional or psychological support [20]. We use the term AI-CA to refer only to hybrid and generative (LLM-based) AI agents with therapeutic intent; where evidence concerns the generative AI (eg, ChatGPT [Open AI]) specifically, we use the term generative AI-CA. While evidence on AI-CA in mental health care remains limited overall [17,21], research specifically addressing its use in BPD or within DBT frameworks is, to our knowledge, absent. This may reflect iatrogenic-risk concerns and the ethical, legal, and practical challenges of managing self-harm and suicidality in this population [22,23].

How patients and professionals perceive AI-CA is central to responsible implementation, as multiple factors at both the patient and provider level shape the acceptability and adoption of conversational agents in health care [24]. Several studies have examined how users perceive generative AI-CA and AI-CA for mental health support [25-28]. Common findings indicate that AI-CA are perceived as accessible, helpful, and nonjudgmental, but also limited by a lack of emotional depth, difficulties with complex situations, and an inability to actively guide the therapeutic process. Clinicians, in turn, tend to favor administrative uses of AI, such as documentation, while showing uncertainty toward client-facing applications, though openness may increase with greater exposure [29].

### Potential AI-CA Integration in DBT: Opportunities and Functional Risks

The relevance of AI-CA to DBT cannot be evaluated only by asking whether AI can produce empathic, coherent, or clinically plausible responses. DBT therapists respond strategically, reinforcing skillful behavior; that is, responding so that the behavior becomes more likely to recur. Whether a behavior is skillful is not given by its topography (the observable form of the behavior, described independently of its consequences) but by its function (the effect it produces in a given context) [9,11]. Determining this requires attention to the context, frequency, and duration of the behavior, to the patient’s current capabilities, and to the patient’s short- and long-term goals. Calling for support during an intense urge to self-harm is skillful for a patient who cannot yet tolerate the emotion alone. The same call becomes a safety behavior when the patient is no longer seeking coaching but repeated confirmation that nothing bad will happen. Safety behaviors are actions taken to prevent a feared outcome that is in fact unlikely; they relieve anxiety in the moment, which maintains them, while preventing the patient from learning that the feared outcome does not follow [30].

Two features of AI-CA are relevant against this background. First, generative AI-CA tends toward sycophancy, affirming users’ statements and self-image rather than challenging them, a tendency linked to reduced prosocial intentions and increased dependence [31]. An agent that responds affirmingly to whatever the patient brings may therefore reinforce what treatment targets for change, for example, by supporting avoidance where exposure is indicated, or by confirming a patient’s belief that they cannot cope. The concern is not merely hypothetical: studies of suicide-related AI responses show inadequate crisis responses, limited contextual understanding, and a need for safety evaluation [22,32]. Second, unlike human therapists, AI-CA respond immediately and on demand. This lowers the threshold for seeking support, which may aid skills generalization but may equally sustain safety behaviors and reduce opportunities to tolerate distress or learn skills more independently. AI-CA have also been found to facilitate self-disclosure by reducing perceived fear of judgment, although in nonclinical samples [33]. How a constantly affirming and always-available agent affects patients who already may struggle with relationships, boundaries, and expressing their own wants and feelings remains an open question.

There is a need for understanding how AI-CA might be responsibly integrated into DBT, particularly given limited access to specialist BPD treatments such as DBT [2] and the potential for AI-CA to support between-session skills practice and generalization. However, methodological guidance on how to integrate AI into psychotherapy remains underdeveloped [7], and the perspectives of method-specific clinicians who would be responsible for such integration have received limited attention. Because the adoption of AI-enabled health care tools depends on stakeholders’ perceptions and acceptability [24], qualitative methods are well suited to capture how clinicians make sense of risks, responsibilities, and fit within clinical practice. Since AI integration in DBT is not yet established, participants’ accounts are treated as anticipatory and interpretive rather than experiential reports of implemented practice. Such expectations are analytically relevant because emerging technologies are shaped not only by technical capability, but also by professional imaginaries, anticipated risks and benefits, and role negotiations before implementation occurs [34]. Therefore, this study aims to explore how DBT clinicians in Sweden understand the anticipated integration of AI-CA into DBT for individuals with BPD, specifically regarding the relational positioning of AI-CA and perceived suitability of DBT functions or tasks for AI-CA involvement.

## Methods

### Study Design

This qualitative study used semistructured interviews to explore DBT clinicians’ expectations about the imagined integration of AI-CA into DBT. Data were generated and analyzed using reflexive thematic analysis (RTA) within a constructivist epistemological framework [35], in which knowledge and meaning are understood as actively constructed through the researcher’s engagement with the data, rather than as preexisting patterns awaiting discovery. RTA within a constructivist framework was chosen because the knowledge sought is interpretive rather than observational. The first author’s dual positioning, as a DBT clinician and as a developer of an AI-CA prototype, was treated as an analytic resource rather than a bias to be neutralized, enabling recognition of treatment-specific meaning in participants’ accounts. Consistent with a constructivist RTA, the findings are understood as constructions shaped through dialogue between participants’ accounts, the interview framing, and the research team’s clinical and theoretical positioning [35]. The study is reported in accordance with the Standards for Reporting Qualitative Research (SRQR) checklist (Checklist 1)[36].

### Ethical Considerations

This study was approved by and followed guidelines from the Swedish Ethical Review Authority (Diary Number: 2024-03541-01). All participants gave written informed consent, participated voluntarily, and did not receive compensation. To protect privacy, data were pseudonymized, removed identifiers before publication, and stored securely by the research team in compliance with the General Data Protection Regulation (GDPR).

### Participant Characteristics and Context Setting

Seventeen psychologists were purposively recruited by emailing DBT teams in publicly funded psychiatric outpatient services in Västra Götaland and Skåne, 2 of Sweden’s largest health care regions, serving approximately 3.2 million inhabitants combined. Inclusion criteria were at least 1 year of experience working with DBT and being a licensed psychologist. In Sweden, psychologist licensure requires a 5-year MSc in Psychology and 1 year of supervised clinical practice. DBT training typically comprises about 10 days over 1 year, considered the minimum team-based standard for delivering DBT in Sweden. Fifteen of the seventeen participants worked in standard 4-mode DBT programs; the remaining 2 in programs without telephone coaching. Participant characteristics are summarized in Table 1.

**Table 1.** Participant characteristics (n=17).

Characteristics^a^	Values, n (%)
Age (years)	
20‐29	2 (12)
30‐39	4 (24)
40‐49	6 (35)
50‐59	4 (24)
≥60	1 (6)
Gender	
Women	13 (76)
Men	4 (24)
DBT^b^ experience (years)	
1‐3	7 (41)
4‐6	4 (24)
7‐9	2 (12)
≥10	4 (24)
AI-CA^c^ usage	
Daily	2 (12)
Weekly	8 (47)
Monthly	2 (12)
Rarely	1 (6)
Never	4 (24)
Self-rated computer skills	
High	4 (24)
Medium	12 (71)
Low	1 (6)
Attitude toward AI	
Positive	5 (29)
Neutral	3 (18)
Negative or concerned	2 (12)
Mixed	7 (41)

a Participant characteristics were self-reported using predefined response categories (Multimedia Appendix 1).

b DBT: dialectical behavior therapy.

c AI-CA: artificial intelligence conversational agent.

### Procedure

The interview guide provided a common structure oriented toward DBT-specific implementation questions, including relational positioning, responsibility, treatment functions, and BPD-specific concerns (Multimedia Appendix 1); it was used flexibly, with follow-up probes pursuing clinically and analytically salient issues. Attention to anticipated AI-CA capacity to understand reinforcement contingencies developed early, after participant 3, and informed subsequent interviews. Participants were asked to consider a not-yet-implemented, DBT-integrated AI-CA whose autonomy and role were left open for them to position themselves.

The mean interview length was 51 (SD 9.9; range 36-80) minutes. The participant group comprised 13 women and 4 men, with a mean age of 43 (SD 10.3, range 24-60) years and a mean of 6.4 (SD 5.6; range 1-18) years of DBT experience. No participant dropped out after agreeing to take part. One pilot interview was conducted to refine the interview guide and was included in the study. Three participants from the first author’s DBT team were included but interviewed by coauthor LA, who has no connection to the participants, and the rest by the first author. Interviews were conducted via secure video (12 of 17) or, when it was possible, in person from January 28 to June 3, 2025, at the workplaces of participants. Information power was assessed reflexively throughout the process, and the 17 interviews provided rich and diverse data that enabled deep engagement with the research questions [37]. This decision regarding the number of participants relied on the study’s narrow scope (AI integration in DBT), the highly specific participant group (DBT psychologists), and the rich quality of the dialogue, although no established theory was applied and the analysis was cross-case, which may have lessened the information power of the participant group. Recruitment was guided by information power rather than data saturation and ended when the focused aim, specific participant group, and rich interview material were judged sufficient to address the research questions. Data saturation was not applied, in accordance with RTA methodology [38].

### Data Analysis

The analysis followed Braun and Clarke [35] 6-phase framework. The process was recursive, moving back and forth between phases as patterns of meaning were developed and refined. The first author conducted the primary analysis, with 2 team discussions to explore and challenge interpretations of codes and themes, and more frequent analytic consultations with supervisors (SS, LA, and SIL). Analytic discussions functioned as critical reflexive dialogue to challenge and deepen interpretations rather than to establish coding consensus. The initial focus on power, responsibility, and potential iatrogenic effects was gradually refined into a more nuanced concern with relational dynamics and the difficulty of determining the function of AI-generated responses within a therapeutic context. Later stages involved refining, defining, and naming themes, and then producing the final report.

The first author became familiar with the data by underlining transcripts and listening to each interview at least 3 times. Each iteration clarified the rationale for asking certain questions while omitting others, for example, questions concerning the management of suicide risk. Early analytic coding used Post-it notes and mind maps, which were iteratively clustered into candidate patterns of meaning (see Multimedia Appendix 2 for process documentation). Visualization made it easier to see overarching patterns of meaning and form themes. Later, NVivo 15 (Lumivero) was used to manage the dataset; however, final coding and theme development were conducted in a spreadsheet format, as this afforded greater flexibility for the recursive, interpretative work of collapsing, splitting, and renaming codes in line with RTA. We did not aim for coder consensus or interrater reliability. A reflexive diary was also used, primarily before and after interviews, to document affective responses such as discomfort or enthusiasm and to help distinguish researcher reactions from participants’ meanings.

Generative AI tools (ChatGPT, Claude) were used only for language editing and manuscript organization, not for data analysis, coding, or theme development; no interview excerpts or participant material were entered into them. Two authors (SS and LA) have previously coauthored work on a separate mental health chatbot prototype (BETSY) for non-DBT contexts [39,40]. This shared research trajectory likely sensitized the analytic dialogue to questions of codesign, feasibility, and acceptability of AI-CA, and was kept in view during reflexive discussions. All interviews were transcribed verbatim in Swedish by the first author. Analysis was informed by the first author’s clinical training in DBT and undergraduate grounding in both psychodynamic and cognitive behavior theory. Behavioral-theoretical concepts such as reinforcement contingencies, the dialectical tension between acceptance and change [9], and relational or projective sensitivities functioned as interpretive lenses rather than deductive frameworks; no predefined codebooks were used.

In line with RTA, the first author’s positioning was considered throughout. A licensed psychologist with over a decade of DBT experience, including work with suicidal patients, the first author is also developing a noncommercial AI-CA prototype within the doctoral project. This dual role, as clinician and as developer of the technology under study, sensitized the analysis to questions of risk, clinical responsibility, and implementation constraints; a fuller reflexive account appears in Multimedia Appendix 2.

## Results

### Overview of Themes

The results are organized around three main themes (Table 2): (1) “Who Are We in Therapy?,” on the relational positioning of AI; (2) “The Stoic Helper,” on functions seen as suitable for AI support within DBT; and (3) “The Well-Intended Accommodator,” on the difficulty of reliably determining whether an AI response supports change or reinforces maladaptive behavior (AI functional ambiguity) and the dependency and responsibility concerns this raises. Across themes, participants often held conflicting views, moving between confidence and uncertainty about whether and how AI should be implemented. Throughout, “AI” refers to AI-CA as defined above.

**Table 2.** Overview of themes from reflexive thematic analysis of dialectical behavior therapy therapist interviews regarding AI integration (n=17).

Themes	Description
Theme 1: Who Are We in Therapy?	This theme focuses on relational positioning rather than technical capability, examining how AI is defined in relation to therapist and person in treatment. Explores efforts to define AI-CA^a^ as a tool, subordinate assistant, team member, or a more expert role.
Theme 2: The Stoic Helper	Participants described AI as able to extend DBT^b^ support beyond ordinary therapist availability, including skills generalization, psychoeducation, homework support, and telephone coaching. Analytically, these accounts were interpreted as positioning AI as a “stoic helper”: available, competent, adaptable, and tireless.
Theme 3: The Well-Intended Accommodator	This theme captures concerns that AI-CA may reinforce dependency, function as a safety behavior, and accommodate distress in ways that undermine autonomy, and the difficulty of reliably determining this (AI functional ambiguity). It also highlights tensions around responsibility, control, and clinical risk.

a AI-CA: artificial intelligence conversational agent.

b DBT: dialectical behavior therapy.

**Figure 1.**
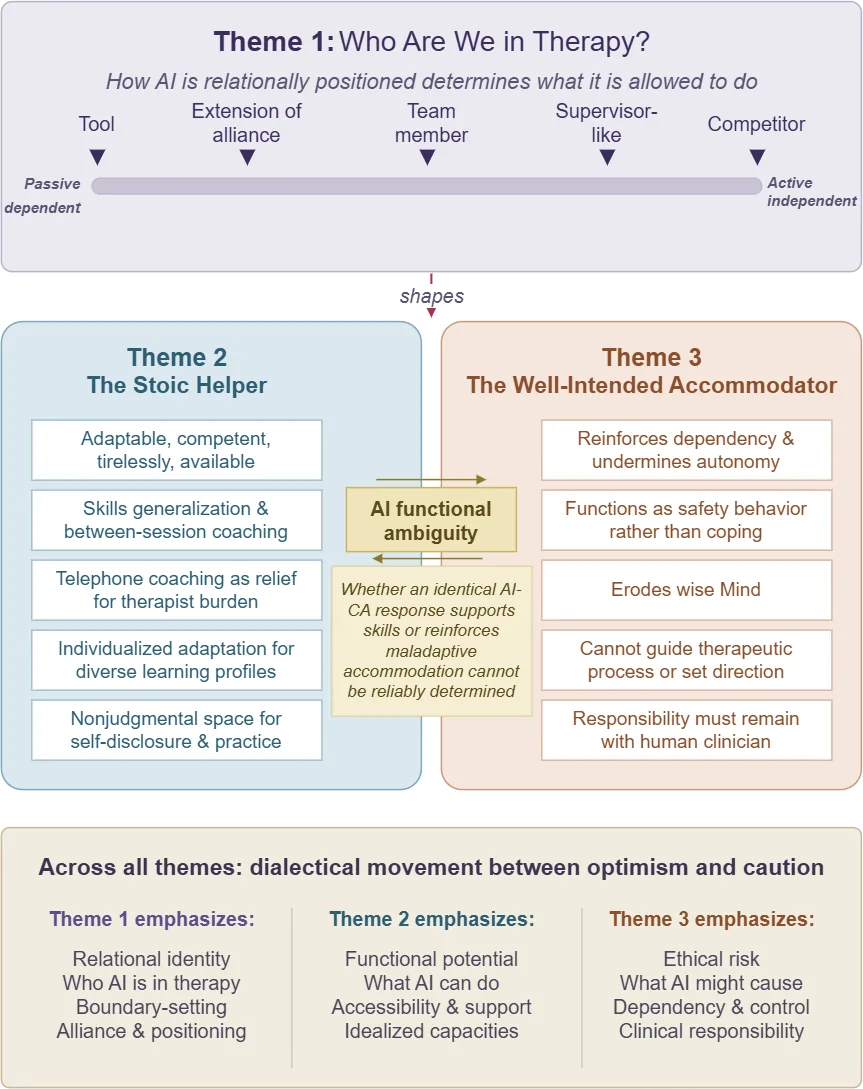
The overview should be read as an interpretive synthesis of relationships developed through analysis, not as a formal explanatory model or empirically tested structure (n=17). Theme 1 operates as a framing condition that shapes how themes 2 and 3 are understood; Themes 2 and 3 form a dialectical pair linked by AI functional ambiguity.

Taken together, clinicians’ views were structured by relational positioning, functional hopes, and ethical concern rather than by simple approval or rejection. Figure 1 presents a conceptual synthesis: theme 1 frames themes 2 and 3, which form a dialectical pair in which the same feature that makes AI a “stoic helper” can make it a “well-intended accommodator,” the juncture at which functional ambiguity arises. These positions sometimes coexisted within a single account, for example: P4 hoped AI could relieve after-hours telephone coaching yet feared it might reinforce a patient’s harmful “ways of thinking,” while P8 saw AI as a possible “bridge” for relationally avoidant patients yet doubted it could grasp the function behind their words. The three themes were developed from recurring patterns across interviews, not from single cases. However, some positions were more widely shared than others. Across interviews, participants commonly discussed availability, boundaries, skills support, roles, dependency, and responsibility. More specific ideas, such as AI-CA as a supervisor or competitor, were less frequent and are presented as illustrative examples rather than prevalence claims.

### Theme 1: Who Are We in Therapy?

This theme centers on relational dynamics rather than technical capabilities, exploring how AI is defined and positioned in relation to both therapist and person in treatment, and which relational capacities it may or may not possess compared with human therapists.

The availability and relational aspects of AI are problematized in terms of what they do to the therapeutic relationship, and that unlimited access is not always an asset or necessarily desirable.

If the patient has unlimited access to an AI and chats with it many hours a day, in a way that [she,he or they] doesn’t with me. And if that AI also acts like another person, like, or like a therapist. Then there is, you know, a risk... Then I see it as that… Then the alliance between me and the patient would, you know, would be weakened based on it becoming stronger with the AI. And it’s not certain to me that a patient who has personality disorder and a tendency for idealization and splitting... It’s not certain that such a patient would see it as that the AI is just a complement to me.

[P16]

Expanding on this, a participant reflected on the universal human need and capacity for relationships, attachment, and forming relational bonds; “we are made to form relationships. I mean, I just ask ChatGPT about rabbit poop and I want to write thank you. Even though I get that there is nobody to thank” [P5]. Participant [P17] takes this somewhat further by considering what this relational capacity may mean for the individual who forms an attachment: “I think that the risk that it [AI] is experienced as supportive without being there” [P17]. These accounts are read as suggesting that AI can evoke relational attachment patterns, for both patients and therapists alike, where boundaries between therapeutic and personal relationships are less clear.

AI’s position in relation to the therapeutic frame shaped what it was seen as allowed or expected to do, with participants describing AI variously as a tool, a team member, or as having the characteristics of a supervisor.

In DBT as I see it like I am team therapist who treats a group of patients. It’s not just me and the patient in the treatment relationship and that there are many people [therapists] who can help each other out, think and do skills training together and things like that. That it’s a community, like. I think that getting additional help there from an AI, if you use it in the right way, then I think the chance that it gives much more benefit than it would harm, like.

[P3]

Given that DBT is a team-based treatment, several participants suggested that AI could be understood as part of that team, also suggesting that in some respects AI may help therapists make better decisions, much like a supervisor would.

If you imagine far ahead in the future then maybe an AI could be part of [the team] and reason with the therapists. The therapists can, you know, spin off and be emotion-driven.

[P14]

How AI is positioned here also shapes which qualities it is expected to possess, those a good colleague or supervisor would need in order to perform such tasks.

Many participants described availability as important for patients. However, this participant points to how it could also matter for therapists. This account is interpreted as suggesting that, from a therapist’s standpoint, the risk of fostering dependency is less obvious and is instead framed as a question of whether AI’s competence can be “trusted.”

Sometimes it has been urgent and then we have tried to reach our external supervisor. But he might not always be available. So that in those more urgent situations I think that even when it comes to urgent assessments of people with suicide risk… And at the same time, I don’t really know. It’s also this thing, should you then trust an AI bot for those important judgments? But I guess it’s when you need it urgently and there is no one else available that you can turn to.

[P4]

One way of understanding this is that there is an ambiguity around what AI could or should do. AI was also attributed considerable competence here, alongside a skepticism about even entertaining that possibility. On the other side of the relational spectrum, some participants felt more comfortable with a more passive or dependent AI that carried out tasks delegated by the therapist, like an assistant.

[AI could] be kind of like information gathering, really, and collect information in some way, like the use of diary cards [a daily self-monitoring tool in DBT] roughly. So that you don’t have to spend that time on this in the session room.

[P15]

But somewhat later in the interview the participant expanded further:

Not in direct patient contact. But more like an indirect part of your work. To follow... Follow the patients passively. More from the outside. More like a surveillance. Not surveillance. But someone who keeps an eye on things, not in direct contact really. So like, that AI could be helpful for the therapist.

[P15]

These accounts can be read as AI being easier to construct as less threatening when positioned as a tool or more dependent on the therapist, which may function as a response to perceived uncertainty and a sense of losing control and ambivalence regarding the role or function of AI in therapy.

Several participants constructed AI as something that should have boundaries in its contact with the patient, like those of a human therapist or team member.

If the behavior is too destructive, I think that an AI also should do that [set limits]. [AI responds to a patient] “I get that you are angry now, but you can’t say that to me, you can’t talk like that.”

[P16]

In this example, the participant accepts that AI is ’setting a boundary’ with the patient, which can be read as positioning AI as more than a tool, as something closer to having a subject. This could be interpreted as making two claims at once: that AI is something to be respected, or that patients with relational difficulties need to treat AI ’as if’ it were a person in order to learn new behaviors.

Below, a question is raised about what happens when, or if, the trust between AI and patient is high.

Because it gets kind of weird if the patient starts trusting an AI bot more than they trust the therapist. It gets kind of weird with... I mean the alliance is affected, and the trust in the therapist. Patients may feel that they get better advice that fits me more, that AI can adapt more than my therapist.

[P4]

The therapist and AI are situated as competing for the patient’s trust on the basis of perceived competence and adaptability, with the therapist being diminished within the therapeutic relationship.

A participant reflects on the timing of AI introduction, similar to participant [P2], suggesting that the therapist and patient may first need space to establish a relational connection before AI is incorporated into therapy: “In the orientation phase when you are supposed to form some kind of alliance and trust. All of those parts feel very strange that you would bring in AI there” [P11]. The participants frame the solution as establishing a therapeutic alliance before introducing AI, without in this instance, considering that the therapeutic relationship often fluctuates over the course of therapy.

This theme addresses how AI is relationally defined and where its boundaries are drawn, which shapes what participants saw as possible in team-based DBT.

### Theme 2: The Stoic Helper

AI was understood as “a stoic helper” that was able to provide support at times when human therapists either could not or preferred not to be present, such as after hours or on weekends. More concretely, the stoic helper was exemplified by participants in performing difficult-to-sustain therapeutic DBT functions. These could be skills generalization, psychoeducation, help with homework, and between-session availability and telephone coaching. When AI is described as performing these functions, it is interpreted as implying that AI would possess characteristics of being available, competent, adaptable, and tireless.

Many participants thought about telephone coaching:

I immediately thought about this thing with the phone coaching and crisis management. That it maybe could be a really good idea to be able to talk to an AI therapist when there’s a crisis.

[P7]

Here it is understood that AI could become an “AI therapist,” with less focus on why the patient should not call the human therapist, or what consequences calling AI instead might have. The next excerpt highlights telephone coaching as a burden, while also conveying hopes that AI could share some of this load. “In DBT treatment I probably perceive it as a good complement. Maybe even some form of relief regarding the phone coaching specifically” [P16]. Expanding on the capacity to stay with the patient in a wider range of ways: “that it [AI] doesn’t get tired and stuff, that is a possible advantage. Some patients maybe need to hear something very many times” [P14]. AI is interpreted as possessing therapeutic qualities in areas where human therapists may sometimes fall short. Neither participant acknowledged that they themselves might become dysregulated during telephone coaching. This participant further reflects on telephone coaching: “To use as phone coaching for example when we are not available. Because it’s often at night that they can need phone coaching and then we are not available” [P4], interpreted as expressing a therapeutic wish to be present in moments when human availability is inevitably limited and needed.

Below, expanding on contacting AI instead of the human therapist, the contact between therapist and patient is not itself problematized. Instead, AI is positioned as a possible solution to what could be constructed as a shared problem within the therapeutic relationship.

There are quite many who don’t want to bother their therapists [doing telephone coaching]. Who don’t want to burden by reaching out, whether it’s in the evening or daytime or whatever. They don’t want to reach out to the mobile team, they just don’t want to.

[P2]

Analytically, the participant can be interpreted as hoping that a frustration in therapy, patients not reaching out, could be addressed by introducing an AI-CA that would always be available and could not be burdened. Expanding on this further, the next participant mentioned: “Sometimes we give really super complicated homework assignments, I think. And then the patients just stand there and just, “I have no idea [how to do the homework]” [P12]. AI was interpreted as competent and benefiting not only patients but also therapists, as the examples participants referred to often concerned situations they themselves experienced as difficult to manage. These accounts framed AI as a response to patient needs and pointed to strain points in DBT delivery that clinicians may experience as difficult to sustain, without directly relating this to their own limited resources.

Another participant reflected on AI’s relational capacities, interpreted here as a concern that AI might compete with the therapist for the therapeutic relationship, which needs to be protected.

I mean, I think you maybe don’t introduce it right away. You maybe do it when you feel that you have an alliance and you like can almost have it as a kind of extended arm of the therapeutic alliance, then maybe I don’t think it can affect [the alliance] that much.

[P2]

However, this is understood as suggesting that, if introduced the “right” way, AI could be seen as aligned with the therapist, an extension of the therapy rather than a separate, competing entity.

A substantial component of DBT involves skills learning and generalization. The nonhuman aspects of AI were in this account read as being beneficial since it removes a layer of relational complexity for some patients.

Maybe you are a person who likes to learn in other ways, in some digital way, then maybe it can be an aid. Exactly, if you have someone who has quite a lot of relational deficits or a lot of anxiety maybe, like social anxiety, then it could have been a bridge into something.

[P8]

AI was interpreted as more adaptable and competent than human therapists and could provide a higher degree of individualization when teaching skills in treatment, and points to that AI is experienced differently than a human therapist.

This participant’s account expands on skills training further and could be read as expressing frustration with limits within the therapy setting and the potential of AI in this regard.

I know that some patients leave the skills training and then they experience that they didn’t understand the skill even though you asked them if there is anything that’s unclear or maybe I am not always completely clear[when giving instruction], so they leave and then maybe there is an experience of this isn’t going to work or I don’t understand this. Then AI can help them a bit more in a different way.

[P4]

AI is interpreted as having capacities distinct from those of human therapists and as compensating for therapist difficulties, or as being better positioned to teach skills because it is not constrained by time or group-based settings.

How a therapeutic situation can be understood or analyzed varies by its nature, and more than one answer can be “right.”

As long as it’s not something that would encourage the patient to do something destructive. A situation can be handled in many different ways, without my answer needing to be right or the AI’s answer needing to be right. But it’s probably actually a good thing that there are several different answers to one and the same situation.

[P10]

The participant is interpreted as suggesting that AI could broaden the perspective in therapy and give the patient more options rather than just listening to the therapist’s voice.

This theme addresses AI’s anticipated functions in DBT and the opportunities its accessibility offers, often at points clinicians find difficult to manage but essential to treatment.

### Theme 3: The Well-Intended Accommodator

Participants raised concerns that on the surface helpful AI behavior may reinforce maladaptive behavior, foster dependency, and operate as a safety behavior (well-intended accommodation).

The boundary between helpful support (such as skill generalization) and maladaptive accommodation is difficult to determine in practice as understood in this account:

[If] you expand the possibilities then there is a risk that DBT is counteracted by having an external auxiliary ego [Translator’s note: 'hjälpjag’ is a Swedish therapeutic term referring to an external support that functions as an extension of the patient’s own coping capacity], so that you don’t internalize the skills in yourself. And what happens then if you lose the auxiliary ego?

[P5]

The same participant further reflected on technology in a broader societal context and what skills we might lose or not learn or internalize:

How long we can be bothered to dig into our inner selves and find an answer of our own. I think that’s obvious. I can barely read books anymore without reaching for the phone.

[P5]

While also situating themselves within the same context as the patients, but more on a personal level, not professional. Instead of auxiliary ego, another participant used external knowledge, which here is understood as constructed as the same concept:

It [AI] becomes a bit like it replaces internal knowledge, that it instead becomes some kind of external knowledge, and there’s a risk in that you yourself no longer know it.

[P10]

Here AI is positioned more as something “outside” therapy, hindering learning or growth instead of helping or facilitating it as seen in theme 1. Not mentioned is that the therapist is also external to the patient and someone you could become unhealthily dependent on.

Several participants raised the concept of Wise mind [9,10], which is constructed as the opposite of an external auxiliary ego and connects the effects of AI to the ability to trust one’s sense of inner wisdom or certainty.

You check it yourself [AI answer]. How does this fit with what will be good for me? There is a lot in knowing something with confidence. That I know what will be good for me. That I don’t change something because someone else thinks so. Many [with BPD] find it very hard with setting boundaries and having inner certainty. Is this in line with what I want and what will be good for me? To go against your feelings sometimes and sometimes listen. There is something in this when you try to help [your patients] towards a kind of independent life also, identity and those parts.

[P11]

The participant reflects on how AI might influence our inner wisdom, interpreted here as a concern about how AI may shape identity, particularly for individuals whose sense of self is more vulnerable to external influence, which AI may have considerable capacity to exert.

Here, the participant highlighted both the potential benefits and drawbacks of increased accessibility.

Patients can take more [of their] responsibility for the treatment and generalize the skills better and the risk is, I guess, that you rather become irresponsible. I can notice that myself too when you use AI a lot. That you like don’t even have the energy to go to Google. No, exactly. Then you don’t need to learn it because it’s there anyway.

[P1]

This participant is understood as both pointing to tension between risks and benefits and situating themselves within the same sphere of vulnerability as the patient, similar to [P5]. Also read in this account is the notion of responsibility and using AI to learn or using AI instead of learning.

The next participant reflected on what therapeutic work involves and how AI currently seems to operate, thereby indirectly raising questions about its potential consequences.

AI must know how to respond to what the patient says. It can’t just mirror it, often it’s about [as a therapist] doing the opposite. Knowing when to validate something and when to block or be irreverent or what to reinforce and what not. So, it’s not just about letting yourself be shaped by what the patient says but also shaping what the patient does.

[P3]

The participant is understood as pointing to therapeutic qualities seen as problematic in AI, while also suggesting that patients should, to some extent, be directed within the therapeutic process, not just led. The notion of mirroring could here be viewed as being sycophantic, and therapeutic solutions are given instead of being sycophantic within treatment, which AI currently might struggle with doing.

Here, P4’s reflections are understood as similar:

And if you then use it as a suicidal person and a person with self-harm behavior maybe teach it [AI] your own ways of thinking then maybe it also starts encouraging certain behaviors that are not good for you but that you yourself maybe are positive towards.

[P4]

In these accounts, both AI and patients were interpreted as limited in their capacity to know what is needed therapeutically, whereas the therapist was constructed as the one better able to determine this.

Here, the participant articulates that a therapist does not merely listen to what the patient says but attends to the function it serves within a specific context.

I think that’s where this fingertip feeling [Translator’s note: fingertoppskänsla, a Swedish expression denoting clinical intuition] comes in, and what I know about the patient and previous behaviors. Because I think it’s not always so that you respond to what a patient asks. No, I mean, well yes. And that maybe that’s not the purpose either. But that you help them in other ways to see things. I mean I’m just thinking about how, there might be something else behind it, that the patient themselves can’t put into words or is aware of.

[P8]

The participant questioned how effectively AI could accomplish the task of determining the function of behaviors, given its potential inability to challenge the patient or move beyond the surface of the written text, which expands on P3’s mirroring or P4’s concerns about AI learning to promote harmful behaviors.

Furthermore, another participant is understood as expressing concern about affirming responses or beliefs that, although understandable, do not fit the situation or work against the person’s therapeutic goals.

And that it is [AI] too good at validating the invalid and confirming the patient in what they say... I think that you still need input from other people, when that input is constructive or that when other people don’t agree with what you think and feel. It’s a huge part of therapy and be questioned and reality-tested and get help with that part.

[P3]

Taken together, these accounts suggest an anticipated concern that the same AI response may be experienced as validating in one context yet invalidating in another, and participants did not expect AI to reliably distinguish between the two.

Furthermore, participants expressed concerns about how responsibility for treatment would be managed.

I know myself how I do things and what it looks like, the thing that I do. Or that it didn’t turn out so well, so that’s why I repaired it. There’s something in that feeling that fundamentally needs to be connected to a responsible clinician, that there’s someone there behind it. Or it can feel like you lose control.

[P11]

The participant is understood as contemplating how ruptures might be handled in therapy when an AI-CA is involved, perhaps more so when working with suicidal clients. The next participant is also read as implying that contact with patients is not predictable and you need to be able to adjust and that therapists, in the end, need to maintain oversight, since AI cannot be trusted in this context, “I think that’s hard to hand over this [suicide assessment] to an AI because it’s so much... tone of voice and like knowledge of patients and experience as a therapist of many similar situations and stuff. I think that’s hard to teach to an AI” [P14]. This could also be exemplified with “You still need a person who can check what it says” [P15]. All these accounts are read as concerns around how AI-CA responds in high-risk situations.

This theme concerns challenges to DBT adherence and whether AI-CA can address BPD-specific considerations, particularly their ability to understand patients’ behavior in clinically complex contexts.

## Discussion

### Principal Findings

This qualitative interview study of DBT psychologists’ perspectives on AI-CA developed three themes: Who Are We in Therapy? (theme 1), The Stoic Helper (theme 2), and The Well-Intended Accommodator (theme 3). Theme 1 examined how participants relationally positioned AI-CA, which in turn shaped how they imagined its practical integration into DBT. Theme 2 reflected how clinicians imagined AI-CA as compensating for strain points or helping in DBT delivery. Theme 3 highlighted an anticipated central implementation challenge, AI functional ambiguity, that is, the difficulty of determining whether an AI-CA response is helpful or harmful in a specific context. Taken together, our analysis suggests that participants’ views of AI-CA appeared closely tied to their understanding of their own role and to the boundaries of what AI-CA should or could do. Figure 1 is a conceptual overview intended to reflect how these themes relate to each other. In the figure, how AI-CA is positioned relationally (theme 1) shapes what it is expected to do (theme 2) and the risks this raises (theme 3); themes 2 and 3 are linked by AI functional ambiguity.

#### Relational Positioning of AI-CA (Theme 1)

Questions of boundaries, roles, what different actors are permitted to do, and who ultimately exercises control become especially salient as AI-CA enters therapy. Participants at times expressed uncertainty about what this anticipated shift might entail, partly in relation to how they understood AI’s basic capacity and whether it should be positioned primarily as a tool or as something more independent, like a team member (see the range of positions in Figure 1).

One possibility is that describing the AI-CA more as a tool may reduce relational risk for the patient, make it less likely to compete with the therapist, and give clinicians more control of the therapeutic process. At the same time, this casts the AI-CA as something other than a human therapist, not credited with the capacity to read between the lines or fully grasp the clinical context. Conversely, when AI-CA is positioned as an always-available team member on a more equal footing in therapy, we interpreted participants’ accounts as pointing to a risk of reinforcing dependency or other maladaptive behaviors. When AI-CA was positioned more as a team member, participants sometimes appeared to feel more threatened, or to have a diminished sense of control, while at other times welcoming AI-CA as a possible third colleague in therapy or an “extension of themselves.” Against this background, the more relational or independent aspects of AI-CA were not always viewed negatively. At times, participants saw value in AI-CA offering alternative perspectives or contributing to dialectical movement within therapy.

Because this positioning shapes what AI-CA is expected to facilitate, while in the process creating problems anticipated to be hard to solve, it may help explain why participants sometimes moved back and forth between themes 2 and 3. It may also help explain the difficulty participants described in reliably knowing whether a given AI-CA response is helpful or harmful, which we term AI functional ambiguity and which links themes 2 and 3.

#### The Stoic Helper (Theme 2)

It is likely unhelpful to view AI-CA solely in terms of the functions it is expected to perform in therapy, without also considering the role it is assigned, particularly in therapies where AI-CA is not expected to function as a stand-alone intervention. This is because perceived or attributed role, function, and capacity should converge for a future treatment to work. Where this convergence is anticipated, the stoic helper may be easier to envision as a genuine helper (Figure 1). Most participants pointed to telephone coaching, practice DBT skills, and more individualized treatment as potentially substantial gains in a future treatment. Precisely because AI-CA is still developing, it may be especially open to projections of what clinicians hope AI-CA might become. Participants appeared to express, for example, a wish for AI-CA to perform difficult, repetitive, or time-consuming aspects of treatment. This may reveal not only what clinicians hope AI-CA can offer patients, but also something about the demands or limitations they experience in their own clinical work. Such a shift may increase efficiency in future treatments and, in some cases, enhance patient autonomy, helping to overcome impasses that might otherwise lead to stalled or unsuccessful treatment. Yet delegating such functions raises questions not only about the therapeutic relationship but also about treatment outcomes, particularly if AI-CA does not function as the stoic helper it is imagined to be. The concern is that working through these difficulties is partly how a meaningful and genuine therapeutic connection is formed and an understanding develops of whether behaviors are helpful or maladaptive.

#### AI Functional Ambiguity: Help or Maladaptive Accommodation (Theme 3)?

The third theme concerns the risks associated with increased accessibility and the introduction of technology that cannot be directly controlled. Participants worried that continuous, low-threshold AI-CA availability could reinforce maladaptive behavior, undermine the therapeutic goal of autonomy, and come to function as a safety behavior.

What makes this issue especially challenging is that the boundary between the stoic helper and the well-intended accommodator may be difficult to identify in practice, for both therapists and patients. The topography of the AI-CA’s response may appear identical in both cases, while its therapeutic function differs. The same therapeutic response may support skills generalization in one context but serve as safety behavior in another. Participants’ accounts raised concerns that AI-CA may not reliably discern which function its responses serve in a clinical context. In therapy, initial helpful therapeutic responses may at times be met with resistance, confusion, or skepticism from patients, yet still move them closer to their goals over time. Distinguishing the function of a behavior from its topography is a core clinical task in DBT [9,11]; what is new here is the condition under which that distinction cannot be reliably made. This condition is clinically consequential because, under such ambiguity, a generative AI-CA does not default to a neutral response: LLMs have been shown to exhibit sycophancy, tending to affirm and agree with the user rather than challenge them [31]. In DBT terms, this means the agent may deliver reinforcement precisely where a clinician would withhold it, strengthening the behaviors treatment aims to reduce.

### Comparison With Prior Work

A qualitative study found that therapists conceptualized AI as a “bridge” or complement to therapy, supporting accessibility and structured tasks rather than the relational core of treatment [41]. In this current study, participants positioned AI-CA in more than a single role. Mapping these accounts onto a behavioral intervention technology continuum [42], they range from therapist-led use with AI-CA support to AI-CA-delivered care under therapist oversight, near but never fully automated use.

When participants imagined an AI-CA in DBT with relational capabilities, they thought this might affect the alliance, a question that predates generative AI [43]. Recent AI-CA studies suggest that users may form alliance-like experiences with AI agents [16,44], and perceived alliance has been linked to greater engagement and symptom improvement [44]. Qualitative research with psychotherapists suggests that integrating AI-CA into psychotherapy raises questions about professional role boundaries, ethical responsibility, and the preservation of the human therapeutic relationship [45]. However, these studies examined the patient’s experience of the AI-CA, rather than how an AI agent affects an ongoing therapist-patient relationship. To our knowledge, direct evidence remains limited on alliance when AI, patient, and therapist are integrated within a psychotherapy model such as DBT.

AI-CA have been proposed to support several parts of the suicide prevention pathway, such as disclosure, risk detection, crisis signposting, and clinician-assisted triage [46], which would be important if AI-CA were integrated into DBT. Responses to suicide-related queries appear to have improved over time [32]. Autonomous risk assessment and crisis management remain insufficiently validated and may be unsafe, especially for high-risk or relationally vulnerable groups [19,22], which several participants in this study raised concerns around. Given the AI-CA’s tendency toward sycophancy and the fact that patients with BPD often present with self-destructive and suicidal behaviors, where it is essential not to reinforce maladaptive behavior, the management of anticipated AI functional ambiguity becomes a central clinical concern.

AI functional ambiguity is related to but structurally distinct from safety behavior, a patient-enacted behavior such as seeking reassurance from an AI-CA. AI functional ambiguity, by contrast, is not a patient behavior but a condition pertaining to AI responses in therapeutic interaction. It is the condition under which it cannot be reliably determined whether a given AI response supports treatment-consistent change processes or inadvertently undermines them. The concerns expressed by participants under “The Well-Intended Accommodator” align with an emerging empirical literature on LLM sycophancy, in which interaction with sycophantic models has been shown to reduce prosocial intentions while paradoxically increasing user trust and willingness to continue using such models [31]. Within DBT, validation is defined as communicating to the patient that their responses make sense and are understandable given their history and current life context, while explicitly not endorsing what is maladaptive [9]. DBT operationalizes this through the therapeutic frame, in which the therapist tries to understand each behavior in the patient’s whole context. A generative AI-CA may instead default to sycophancy, for example, by delivering the reassurance a patient seeks, and so reinforce the patient’s safety behavior. Whereas sycophancy is an AI response whose therapeutic function has already been classified as unhelpful, AI functional ambiguity is the condition in which such classification is not reliably available. What distinguishes the concept is that its concern lies one level higher, at the point of functional classification itself.

### Limitations and Transferability

Clinicians did not test an AI-CA, a deliberate choice: any demonstrated model would quickly become outdated and could steer participants’ narratives, and a brief demonstration would capture only a narrow aspect of AI-CA in DBT. In addition, the interview guide framed AI- and therapist-generated responses as potentially hard to distinguish, an assumption supported by prior research [47,48]. Because the interview framing positioned AI-CA as a potential adjunctive tool or support, and at times as resembling an additional therapist, relational issues such as alliance, role boundaries, and responsibility may have been made more salient during interviews. We therefore treat the themes as reflexively developed interpretations shaped by the dialogue between participants’ accounts, the interview framing, and the research team’s DBT-informed clinical perspective. We collected no standard measures of computer or AI-CA use to characterize the participants. DBT program fidelity and individual therapist adherence were not formally assessed. Some participants’ limited AI-CA experience may also have shaped the range and depth of their reflections.

Transferability should also be considered in relation to the EU AI Act, given that participants were situated within this legal context. To our knowledge, no AI-CA is currently implemented in publicly funded DBT. Under current EU and Swedish legal systems, this appears to be complicated [49], which may have shaped participants’ AI-CA expectations. Another more permissive legal setting might have generated different themes. Swedish survey data describe a public that adopts technology early yet holds cautious, mixed AI attitudes [50], possibly shaping participants’ baseline orientation toward AI-CA. Because the sample was predominantly female and drawn from a caring profession, transferability to more gender-diverse clinician groups should be considered with caution, particularly for findings concerning care, emotional availability, dependency, and AI-CA’s relational role. The themes should not be read as gendered, as no gender-comparative analysis was conducted. The participants’ shared DBT frame of reference is, by contrast, possibly a less context-sensitive frame of reference. Participants worked in large public regional services rather than private settings, likely treating patients with substantial psychiatric burden. Because DBT is team-delivered, a participant group including nurses, social workers, or counselors might have surfaced concerns about liability, professional identity, and scope of practice absent from this psychologist-only group. Purposive recruitment may have shaped which views became most visible, particularly among clinicians with more positive or critical views of AI-CA. While telephone coaching practices can vary across DBT programs and health care systems [3], and most of our participants worked in programs that included telephone coaching (15 of 17), which may have shaped their perspectives.

### Future Research and Implications

Participants stressed establishing a patient-therapist alliance before AI becomes involved. They also emphasized making AI less directive later in treatment, so that responsibility returns to the patient. In our view, this implies that the role of AI-CA may need to vary across treatment phases and with patient need. Greater therapist oversight seems warranted when patients present high-risk behaviors, positioning AI-CA as more adjunctive and less autonomous. Future research should develop methods to examine AI functional ambiguity across roles and therapeutic modalities. A key design and research problem is whether AI-CA responses can distinguish skills coaching from reassurance-seeking, including whether explicit therapeutic context reduces maladaptive AI responses and supports responses that balance validation with change, such as prompting skills use. Research should also examine how patient and therapist can converge on the AI’s role, function, and limitations, and how that agreement may need to be adapted across therapy to what both require.

### Conclusions

In this study, 17 DBT psychologists were interviewed to understand the anticipated integration of AI-CA within a specific treatment context, DBT, and for a clinical population, individuals with BPD. In the accounts we developed, AI-CA was constructed not merely as a tool but as introducing a relational position within therapy that likely needs to be addressed explicitly. The hopes participants attached to AI-CA may be understood as a response to demands within therapy itself; imagined uses appeared shaped not only by technological possibility but also by clinicians’ frustrations, wishes, and unmet needs. In the end, the same difficulty therapists face may also apply to AI-CA, most notably what we conceptualize as AI functional ambiguity. This is the risk of not knowing whether responses such as validation foster dependency, function as safety behaviors, or help patients move forward in treatment. Such a development raises familiar therapeutic questions in a new form, including how to maintain shared goals, adherence to specific treatment methods, and clarity around responsibility.

## Supplementary material

10.2196/98840 Multimedia Appendix 1 Interview guide for the semistructured interviews, including the framing presented to participants, background questions, and the topic areas explored.

10.2196/98840 Multimedia Appendix 2 Statement of the first author’s preunderstanding and reflexive positioning, describing clinical and developer background and its influence on data collection and analysis.

10.2196/98840 Checklist 1 SRQR checklist.

## Abbreviations

AI-CA: artificial intelligence conversational agent
BPD: borderline personality disorder
CBT: cognitive behavioral therapy
DBT: dialectical behavior therapy
GDPR: General Data Protection Regulation
LLM: large language model
RTA: reflexive thematic analysis
SRQR: Standards for Reporting Qualitative Research
