# Supplementary material for: Possible Role and Function of AI Conversational Agents in Dialectical Behavior Therapy for Borderline Personality Disorder: Qualitative Interview Study
Source: JMIR Ment Health. 2026 Sep 25;13:e98840. doi: 10.2196/98840 (PMC13614472; doi:10.2196/98840)
Supplement: Supplementary file 1 [file mental-v13-e98840-s001.docx]

**Interview Guide**

Interview framing

*“Today we will talk about the use of AI digital conversational partners in DBT treatments. By AI digital conversational partners, we mean software systems that use artificial intelligence (AI) to offer guidance and support, or even to simulate therapeutic conversations, as if an additional therapist were present to help patients in their treatment.
You may assume that patients can have difficulty distinguishing between responses from a digital conversational partner and those of a “real” DBT therapist, and that a digital conversational agent can communicate via text or speech.
Our aim is to examine whether and how this technology could complement conventional treatment, rather than fully replacing a human therapist. When we ask questions about conversational partners during the interview, we are referring to AI-based digital software programs.
Do you have any questions before we proceed?”
“First, I would like to ask a few background questions”:*

# Background questions

1. **Demographic information:**

- What is your age?
- Which gender do you identify with?
- Where do you live?
- How long have you worked with DBT?

2. **Experience:**

- What is your experience with dialogue agents, for example ChatGPT or similar tools?
- Predefined categories: daily, weekly, monthly, rarely, never
- How would you rate your digital literacy?
- *Predefined categories: high, medium, low*

3. **Attitudes and beliefs:**

- What are your general thoughts or feelings about chatbots, digital conversational agents, and artificial intelligence (AI)?
- Predefined categories: positive, neutral, negative/concerned, mixed
- Have you had any significant experiences related to digital conversational agents, AI, or chatbots? (e.g., positive or negative experiences; personal anecdotes)
- *Predefined categories: yes, no*
- How do you think AI/chatbots will develop over the next 10 years (overall positive or negative for people)?
- *Predefined categories: positive, neutral, negative/concerned, mixed*

4. **Behavior:**

- How often do you engage with dialogue agents/AI? (e.g., daily, weekly, rarely, never)
- *Predefined categories: daily, weekly, monthly, rarely, never*

5. **Expectations and goals:**

- Are there any specific questions, concerns, or opportunities regarding AI, chatbots, or digital conversational partners that you would like to raise during the interview?

## How do DBT experts in Sweden understand the possibilities and challenges of integrating AI-based conversational agents (AI-CAs) into DBT?

- How do you perceive the possibilities of adding digital conversational partners or AI to DBT treatment?
- Could AI improve any aspects of DBT? If so, which?
- Are there specific patient groups in DBT that might benefit more from AI-CA?
- How do you think DBT therapists would react to increased use of AI-CA DBT?
- What challenges do you foresee in implementing AI-CA in DBT?
- What risks do you foresee in implementing AI-CA in DBT?
- What ethical issues might arise when integrating AI-CA into DBT treatment?
- How do you view responsibility and accountability if an AI-CA were to provide advice that leads to negative consequences?

## How do DBT experts construct the relational positioning of AI-CAs in relation to themselves, patients, and the DBT team?

- How might the “presence” of an AI-CA as an “additional therapist” affect the therapeutic alliance?
- How do you think your role as a therapist might change with the introduction of AI-CA?
- How might your professional identity be affected?
- How might patients’ perceptions of you as a therapist be affected?
- How might trust between patient and therapist be influenced by AI-CA?
- How might the “presence” of an AI-CA as an “additional therapist” influence the treatment process?
- Would you find it useful if an AI-CA provided guidance to a patient during episodes of emotional distress?
- When would such guidance be most useful?
- Are there risks associated with this?
- How might the treatment be affected if AI-CA provides advice that diverges from your recommendations?
- How might the treatment be affected if the AI-CA provides advice that is consistent with your recommendations?
- How could one ensure that AI-CA advice is safe and supports the patient’s treatment in a responsible manner?
- Would you want access to chat logs?

## Which DBT functions do DBT experts perceive as suitable or unsuitable for AI-CA involvement?

- If you had the time and opportunity to “chat” with your patients daily as part of their treatment, what would you choose to focus on?
- How do you think your patients would cope with this?
- What might help to make it work well?
- Which functions, skills, or components of DBT treatment could be improved by adding AI-CA?
- How do you think this would work for: support with homework assignments; teaching skills; conducting chain analyses; completing diary cards; supporting the use of a crisis plan/crisis list; or other functions?
- Are there any functions, skills, or parts of treatment that you think AI-CA should not be involved in?

## How do DBT experts understand BPD-specific considerations when reflecting on AI-CA integration?

- Are there any considerations specific to emotionally unstable personality disorder / borderline personality disorder (BPD) that might influence whether AI should be integrated into DBT treatment?
- How might idealization/devaluation dynamics be relevant in this context?
- What happens if the AI-CA does not understand the patient or does not function as intended?
- Newer forms of AI-CA can be more “personal” and easier to form an emotional bond with. In addition, people with BPD may experience separation difficulties. How do you think people with BPD would manage this?
- How might misunderstandings with the chatbot be handled?
- How might issues emerge at the end of therapy?
- If patients were allowed to keep their AI-CA as part of a maintenance/relapse-prevention plan, how do you think that might play out?
- What could be the potential advantages?
- What could be the potential disadvantages?

## Other

- How willing would you be to use AI-CA as part of your DBT treatments?
- Which factors would increase your willingness?
- Which factors would decrease your willingness?
- What type of support or training do you think would be necessary for both therapists and patients to use AI-CA effectively in treatment?
- How do your own experiences and perceptions of digital technology influence your attitudes toward using AI-CA in therapy?
- Finally, are there any questions or reflections that have not emerged during the interview that you think would be important to discuss further?
- Have your views on AI-CA changed from before to after the interview?
