# Supplementary material for: Possible Role and Function of AI Conversational Agents in Dialectical Behavior Therapy for Borderline Personality Disorder: Qualitative Interview Study
Source: JMIR Ment Health. 2026 Sep 25;13:e98840. doi: 10.2196/98840 (PMC13614472; doi:10.2196/98840)
Supplement: Supplementary file 2 [file mental-v13-e98840-s002.docx]

**Preunderstanding**

I entered this study with over a decade of clinical experience in DBT and a generally positive orientation toward technology in mental health care. This initially oriented me toward identifying practical functions that AI might augment DBT, such as skills coaching and diary card support. As the analysis progressed, I became more attentive to how AI integration might affect relationships, teamwork, and accountability in treatment. My clinical experience with suicidality sensitized me to participants’ concerns about responsibility and harm in possible adverse events, which became a recurring interpretive lens in coding and theme development. In parallel, developing a beta app prototype sharpened my awareness of how design decisions around data flows, security, autonomy, and decision-making shape what AI can do in practice. Over the course of the study, developments in the field also shifted my stance from general skepticism about feasibility toward viewing implementation as increasingly plausible, which likely influenced the later interviews. Developing and testing prototypes made the difficulty of steering and predicting AI responses across scenarios concrete, so that increased feasibility heightened rather than reduced my concern; at the same time I was struck by how clinically plausible AI responses could appear. Holding these two impressions together — surface clinical credibility alongside underlying unpredictability — likely sensitised the analysis to the same tension participants raised, while also creating a risk of over-reading functional ambiguity into more neutral accounts, which I tried to check through the reflexive diary and team dialogue.

This tension between wanting to contribute to the development of an AI conversational agent and remaining acutely aware of suicide risk affected my analysis from the outset. I found myself drawn early to themes such as *responsibility without control* and to framings such as *Can you hold my darkness…?*, drawn from a participant quote.

Relational framings likely entered the analysis early, partly because the interview guide itself departed from relational questions; being aware of this, I deliberately attended to patterns of meaning that the guide did not prescribe, such as participants' functional and ethical reasoning, when developing themes. My interpretive attention was also anchored in a recurring personal standard: if I were ever to implement an AI conversational agent clinically, I would need to be able to account — to patients' relatives and to myself — for why specific design and implementation choices were made. During interviews and analysis, I became increasingly aware of how similar and yet different my position was from that of the participants: in an imagined future where AI becomes part of DBT, we would share a sense of responsibility, though carried differently — mine as a developer and clinician, theirs as clinicians. This recognition of partially shared, partially divergent positionality formed part of the co-construction of the themes.

This reflexive awareness highlighted how my analytic attention was shaped both by participants’ accounts and by my own concerns about risk, responsibility, and harm. The final themes were developed through that interplay.

Photograph of early-phase analytic work: initial codes generated on Post-it notes and iteratively clustered into candidate patterns of meaning. Colors represent provisional groupings that were later developed, merged, and refined.
