# Supplementary material for: Possible Role and Function of AI Conversational Agents in Dialectical Behavior Therapy for Borderline Personality Disorder: Qualitative Interview Study
Source: JMIR Ment Health. 2026 Sep 25;13:e98840. doi: 10.2196/98840 (PMC13614472; doi:10.2196/98840)
Supplement: Supplementary file 3 [file mental-v13-e98840-s003.docx]

**SRQR Checklist**

*Standards for Reporting Qualitative Research*

(O'Brien et al., 2014)*.*

| **Item** | **SRQR criterion** | **Location in manuscript** | **Status and comments** |
| --- | --- | --- | --- |
| **Title and Abstract** | | | |
| **S1** | Title: Identifies the study as qualitative or indicates the approach/method; concisely describes topic | Title page (p. 1) | Addressed. The title explicitly identifies the study as a qualitative interview study and specifies the phenomenon, therapeutic context, and population. |
| **S2** | Abstract: Summarizes background, purpose, methods, results, and conclusions using the target journal's format | Abstract (pp. 2–3) | Addressed. The abstract includes Background, Objective, Methods, Results, and Conclusions, and reports the participant group, analytic approach, main themes, and overall interpretation. |
| **Introduction** | | | |
| **S3** | Problem formulation: Describes and justifies the phenomenon studied; reviews relevant literature; states the problem or gap | Introduction (pp. 4–6) | Addressed. The manuscript establishes the clinical burden of BPD, limited access to DBT, AI-CA, and the gap in treatment model-specific implementation knowledge. |
| **S4** | Purpose/research questions: States the purpose and specific objectives or research questions | Introduction, final paragraph (p. 6) | Addressed. The aims are clearly stated. |
| **Methods** | | | |
| **S5** | Qualitative approach and research paradigm: Names approach; identifies epistemological position; rationale required | Study design (p. 7) | Addressed. Reflexive thematic analysis is named and described, and a constructivist epistemology is explicitly stated with an interpretive rationale. |
| **S6** | Researcher characteristics and reflexivity: Describes researcher background, assumptions, and relationship to topic/participants | Data analysis (p. 10); Multimedia Appendix 2 | Addressed. The first author's DBT experience, technology orientation, work with suicidality, reflexive diary use, and developer position in relation to the topic are reported in the manuscript and expanded in Multimedia Appendix 2. |
| **S7** | Context: Describes setting and relevant contextual factors; rationale for site selection required | Participant characteristics/Context setting (p. 7) | Addressed. The study setting is described as publicly funded psychiatric outpatient DBT services in Region Västra Götaland and Region Skåne, two of Sweden's largest healthcare regions (approximately 3.2 million inhabitants combined). The broader service context is specified, including the Swedish licensure pathway for psychologists, the standard DBT training format, and the distinction between standard four-mode DBT programs and the two programs without telephone coaching. Recruitment was conducted by emailing DBT teams in these services. |
| **S8** | Sampling strategy: Describes how and why participants were selected; addresses criteria for stopping sampling; rationale required | Participant characteristics/Context setting and Procedure (pp. 7–9) | Addressed. Purposeful sampling and inclusion criteria are specified, and information power is used as the rationale for determining the adequacy of the participant group. |
| **S9** | Ethical issues: Documents ethics board approval, informed consent, confidentiality, and data security | Ethical Considerations & Data storage (p. 7) | Addressed. Ethics approval, written informed consent, voluntary participation, pseudonymization, and GDPR-compliant storage are reported. |
| **S10** | Data collection methods: Describes data types, collection procedures, timeframe, and iterative process; rationale required | Study design (p. 7) and Procedure (p. 8) | Addressed. The manuscript describes semistructured interviews, iterative questioning, interview mode, timeframe, and the rationale for using qualitative interviews. |
| **S11** | Data collection instruments and technologies: Describes instruments and recording devices; notes changes during study | Procedure (p. 8); Multimedia Appendix 1 | Addressed. The interview guide with future oriented framing is provided in Multimedia Appendix 1, a pilot interview is reported, and the use of secure video or in-person interviews is described. The manuscript also notes a change during the study: heightened analytic attention to AI's capacity to understand reinforcement contingencies developed after the third interview and informed subsequent interviews |
| **S12** | Units of study: States number and relevant characteristics of participants; indicates level of participation | Participant characteristics/Context setting and Table 1 (pp. 7–8) | Addressed. The manuscript reports N=17, mean interview length, absence of dropouts, and participant characteristics including age, gender, DBT experience, and AI familiarity. |
| **S13** | Data processing: Describes transcription, data management, coding, and anonymization procedures | Ethical (p. 7); Data analysis (pp. 9–10) | Addressed. Verbatim transcription, dataset management in NVivo 15, pseudonymization, and the statement that AI was not used for coding or theme development are reported. |
| **S14** | Data analysis: Describes process for developing inferences/themes; identifies analysts; rationale required | Data analysis (p. 9) | Addressed. Braun and Clarke's six-phase framework is described as recursive rather than linear, the primary analyst is identified, and interpretive team discussions are reported as reflexive dialogue rather than consensus procedures. |
| **S15** | Techniques to enhance trustworthiness: Names and describes techniques; rationale required | Procedure (p. 8) and Data analysis (pp. 9–10); Multimedia Appendix 2 | Addressed. Reflexive diary use, repeated listening, sustained immersion, analytic discussions, and supervisory consultation are described as reflexive supports for analytic depth and transparency within an RTA framework. |
| **Results/Findings** | | | |
| **S16** | Synthesis and interpretation: Presents main findings as interpretations or themes; may integrate prior research or theory | Results (pp. 11–22) | Addressed. The findings are organized into three interpretive themes, supported by a summary table, participant excerpts, and integrative commentary. |
| **S17** | Links to empirical data: Provides quotes or excerpts to substantiate analytic findings | Results (pp. 14–22) | Addressed. Translated excerpts from Swedish interviews are provided with participant identifiers. |
| **Discussion** | | | |
| **S18** | Integration with prior work, implications, transferability, and contribution: Connects findings to literature; addresses scope of application; identifies unique contribution | Discussion (pp. 23–28) | Addressed. The discussion connects findings to adjacent literature and outlines implications, transferability and future directions. |
| **S19** | Limitations: Addresses trustworthiness and specific limitations of findings | Limitations (p. 26) | Addressed. The limitations are specific and methodologically grounded, including the absence of AI demonstration exposure, variation in participant AI familiarity, and the qualitative scope of the account. Also that the interview framing may have shaped participants' narratives, that program fidelity and therapist adherence were not formally assessed, and that the predominantly female sample limits transferability to more gender-diverse groups. |
| **Other** | | | |
| **S20** | Conflicts of interest: Declares potential sources of influence and how they were managed | Conflicts of Interest (p. 29) | Addressed. A potential conflict is disclosed for the first author, who is developing a noncommercial DBT-related AI prototype; the remaining authors report no conflicts of interest. |
| **S21** | Funding: States funding sources and describes role of funders in the study | Funding statement (p. 29) | Addressed. Funding sources are specified and the manuscript states that the funders had no role in study design, data generation, analysis, interpretation, or manuscript preparation. |

**References**

1. O'Brien BC, Harris IB, Beckman TJ, Reed DA, Cook DA. Standards for Reporting Qualitative Research: A Synthesis of Recommendations. Academic Medicine. 2014;89(9):1245-1251. doi:10.1097/ACM.0000000000000388

2. Braun V, Clarke V. Thematic Analysis: A Practical Guide. London: SAGE; 2022.

3. Malterud K, Siersma VD, Guassora AD. Sample Size in Qualitative Interview Studies: Guided by Information Power. Qualitative Health Research. 2016;26(13):1753-1760. doi:10.1177/1049732315617444
